# Mass spectrometric analysis of TRPM6 and TRPM7 from small intestine of omeprazole-induced hypomagnesemic rats

**DOI:** 10.3389/fonc.2022.947899

**Published:** 2022-08-29

**Authors:** Nattida Kampuang, Narongrit Thongon

**Affiliations:** Division of Physiology, Department of Medical Sciences, Faculty of Allied Health Sciences, Burapha University, Chonburi, Thailand

**Keywords:** hypomagnesemia, oxidation, phosphorylation, proton pump inhibitors, small intestine, TRPM6, TRPM7

## Abstract

Disruption of small intestinal Mg^2+^ absorption has been reported as the underlying mechanism of proton pump inhibitor-induced hypomagnesemia (PPIH); hence, this study evaluated the expression, localization, phosphorylation, and oxidation of transient receptor potential melastatin 6 (TRPM6) and TRPM7 in the small intestine of rats subjected to PPIH. The expression and localization of cyclin M4 (CNNM4) was also analyzed. We show that, compared to control rats, membrane expression of the TRPM6/7 heterodimer and TRPM7 was markedly lower in the duodenum and the jejunum of PPIH rats; in contrast, expression of membrane TRPM6 and CNNM4 was higher in these organs. Mass spectrometric analysis of TRPM6 demonstrated hyper-phosphorylation, especially T1851, and hyper-oxidation at M1755, both of which can suppress its channel permeability. Further, hypo-phosphorylation of S141 and the dimerization motif domain of TRPM6 in PPIH rats might be involved in lower TRPM6/7 heterodimer expression. Hypo-phosphorylation, especially at S138 and S1360 in TRPM7 from PPIH rats disrupted stability of TRPM7 at the cell membrane; hyper-oxidation of TRPM7 was also observed. These results help explain the mechanism underlying the disruption of small intestinal Mg^2+^ absorption in PPIH.

## Introduction

Proton pump inhibitors (PPIs) use is associated with an increased risk of gastric and colorectal cancer (1). A previous large population-based cohort study reported that the use of PPIs was associated with a 45% increased risk of gastric cancer compared with the use of histamine-2 receptor antagonists (1). The risk of cancer increased with cumulative duration of PPIs administration (1). Prolong use of PPIs also induced hypomagnesemia and systemic Mg^2+^ deficiency (2, 3). Mg^2+^ deficiency impairs DNA repair mechanisms (3), which then increase digestive cancer risk (4, 5). Dysregulation of gastrointestinal Mg^2+^ channels is proposed as the regulatory factors of digestive cancer cell fates and oncogenic signaling pathways (4). Therefore, PPIs induced risk of gastrointestinal cancer probably due, at least in part, to dysregulation of Mg^2+^ channel.

PPI-induced hypomagnesemia (PPIH) has been described in humans (2), C57BL/J6 mice (6), and Sprague–Dawley rats (7, 8), and we have previously reported that, compared to control animals, prolonged omeprazole injection can markedly suppress small intestinal Mg^2+^ absorption in a rat model of PPIH (7, 8) with up to 81.86%, 70.59%, and 69.45% reduction in absorption at the duodenum, the jejunum, and the ileum, respectively (7). Notably, even though this reduction led to significantly higher expression of transient receptor potential melastatin 6 (TRPM6) and cyclin M4 (CNNM4) in the entire intestinal tract of the PPIH rats (7), Mg^2+^ absorption was not restored in these PPIH rats, and the mechanisms remain unclear. Two common single nucleotide polymorphisms in *TRPM6* gene have been reported in PPIH patients (9), and it is possible that TRPM6 protein dysfunction or mutation occurred in the intestine of our PPIH rat model. Moreover, expression and localization patterns of TRPM7 in small intestine during PPIH remain unknown.

It has been previously proposed that the small intestine absorbs Mg^2+^ solely through an un-regulated paracellular pathway because the *TRPM6* gene was not detected in the murine small intestine (3, 10); however, we have previously demonstrated that the duodenum, the jejunum, and the ileum absorb Mg^2+^ through both transcellular and paracellular mechanisms (7, 8). Further, TRPM6 is markedly expressed in duodenal, jejunal, and ileal tissues in rats (7) and humans (11), and a recent study has reported that fibroblast growth factor-23 and parathyroid hormone systemically and directly regulate both transcellular Mg^2+^ absorption and membrane TRPM6 expression in the duodenum and the jejunum (12).

TRPM6 and TRPM7 are bifunctional proteins and consist of a cation channel segment that is covalently linked to an α-type protein kinase domain. TRPM6 shows tissue-specific expression in the intestine and in the renal tubules (13), and plays a crucial role in intestinal and renal epithelial Mg^2+^ transport (14). Mutations in *Trpm6* lead to lower Mg^2+^ absorption despite hypomagnesemia, along with secondary hypocalcemia (HSH) (11, 15), and physiological intracellular Mg·ATP and Mg^2+^ levels are potent negative feedback inhibitors of TRPM6 channel permeability (14–17). In contrast, TRPM7 has a ubiquitous expression (13, 18), plays a role in the regulation of cellular Mg^2+^ (19), and its activity is also inhibited by intracellular Mg^2+^ and Mg·ATP (18–20). Contrary to the above, one previous study has suggested that native TRPM6 primarily functions as a subunit of heteromeric TRPM6/7 channels (21), which show low sensitivity to intracellular Mg^2+^ and Mg·ATP (17, 22); thus, continuous epithelial Mg^2+^ absorption can occur through the TRPM6/7 channel, regardless of intracellular Mg^2+^ and Mg·ATP concentrations. Mutations at S141 in TRPM6 disrupts both localization and function of the membrane TRPM6/7 heterodimer (23) and are implicated in lower Mg^2+^ absorption in HSH. However, TRPM6/7 heterodimer expression in the cell membrane of the small intestine in PPIH has not been characterized.

Phosphorylation of TRPM6 regulates its channel permeability and α-kinase activity, and mutations in the phosphorylation site at S1754 diminishes TRPM6 α-kinase activity (24). Further, phosphorylation at S1252 induces TRPM6 permeability (25), and autophosphorylation of T1851 residue mediates TRPM6 channel suppression by intracellular free Mg^2+^ and activated C-kinase 1 (RACK1) (16). Mutations at the S138 residue in TRPM7 disrupt its membrane localization (23), while phosphorylation of S1360 residues in TRPM7 facilitate its membrane stability (26). It is known that oxidative stress induces oxidation of the M1755 residue in TRPM6, which then suppresses channel permeability (27). Nevertheless, phosphorylation patterns and oxidation status of TRPM6 and TRPM7 in the small intestine of a rat model of PPIH have not been clearly established.

Given the above unknowns, this study aimed to assess the expression and localization of TRPM6, TRPM7, and CNNM4 in the small intestine of a rat model of PPIH, along with the membrane expression of the heteromeric TRPM 6/7 channel. Additionally, mass spectrometric analysis was used to identify phosphorylation and oxidation in TRPM6 and TRPM7 in this model.

## Methods

### Animals and protein samples

This study was performed in parallel with our previous study and used samples from the same experimental Male Sprague-Dawley rats (7). All experiments were performed following relevant guidelines and regulations, including the ARRIVE guidelines (http://www.ARRIVEguidelines.org), and approved by the Ethics Committee on Animal Experiments, Burapha University, Thailand (IACUC 017/2562). Animals were randomly divided into 3 groups i.e., control, and 12-wk and 24-wk omeprazole injection. Control and 24-wk omeprazole-treated groups were administered subcutaneous sham or omeprazole (20 mg/kg: Ocid^®^ IV; Zydus Cadila, India) injections daily for 24 weeks. The 12-wk-omeprazole-treated group was administered subcutaneous sham injections daily for 12 weeks, followed by subcutaneous omeprazole injection for the next 12 weeks. We have previously established that 12-wk and 24-wk omeprazole injections suppressed plasma Mg^2+^ concentration and induced hypomagnesemia by markedly inhibiting small intestinal Mg^2+^ absorption (7).

Cells from the duodenum, the jejunum, the ileum, and the colon of control and PPIH rats were collected by scraping the mucosal surface with an ice-cold glass slide. Tissue was lysed in cold Pierce^®^ Ripa Buffer (Thermo Fisher Scientific Inc., Rockford, IL, USA) with 10% v/v protease inhibitor cocktail (Sigma, St. Louis, MO, United States) and Halt™ Phosphatase Inhibitor Cocktail (Thermo Fisher Scientific Inc.), sonicated, and centrifuged at 12,000 g for 15 min. Total protein was either subjected to western bolt analysis or separated into cell membrane and cytosolic fractions using Mem-PER™ Plus Membrane Protein Extraction Kit (Thermo Fisher Scientific Inc.).

### Immunoprecipitation

Immunoprecipitation of TRPM6 protein was performed using a commercially available kit (catalog no. ab206996; Abcam, Cambridge, UK). In brief, membrane protein samples were incubated with 1:500 anti-TRPM6 antibody (catalog no. PA5-77326; Thermo Fisher Scientific Inc.) overnight at 4°C on a rotary mixer. The antigen-antibody (Ag-Ab) complex was subsequently incubated with Protein A/G Sepharose^®^ beads for 1 hour at 4°C. The Ag-Ab-beads complex was collected by centrifugation at 2000g for 2 min at 4°C and washed thrice in Wash Buffer by centrifugation at 2000g for 2 min at 4°C. After elution with a Glycine-Tris elution buffer, the immunoprecipitated-TRPM6 (IP-TRPM6) protein was stored at -80°C till Western blot analysis. For mass spectrometric analysis, the IP-TRPM6 protein complex was further concentrated using Vivaspin^®^ 20 centrifugal concentrator (Sartorius Stedim Biotech GmbH, Goettingen, Germany).

### Western blot analysis

Total, membrane, cytosolic, or IP-TRPM6 protein samples were resuspended in SDS-PAGE sample buffer containing dithiothreitol (DTT) and heated for 5 min at 95°C. Samples were loaded, separated on SDS-PAGE gel, and electrotransferred onto a nitrocellulose membrane. The membrane was probed with primary antibodies (1:1000 dilution) against TRPM6 (catalog no. PA5-77326; Thermo Fisher Scientific Inc.), TRPM7 (catalog no. ab729; Abcam), CNNM4 (catalog no. SC-68437; Santa Cruz Biotechnology, Santa Cruz, CA, USA), or β-actin (catalog no. ab8226; Abcam, Cambridge, UK). The membrane was subsequently incubated with 1:5000 HRP-conjugated secondary antibodies as needed (catalog no. ab6721 or ab97110; Abcam, catalog no. AP124P; EMD Millipore), the protein bands visualized by Thermo Scientific SuperSignal^®^ West Pico Substrate (Thermo Fisher Scientific Inc.), and images captured on a ChemiDoc™ Touch Imaging System (Bio-Rad, Hercules, CA, USA). Densitometric analysis was performed using ImageJ for Mac Os X (28).

### In-solution protein digestion

For each sample, 20 μg protein was reduced with 100 mM DTT in 100 mM TEA buffer at room temperature for 30 min and alkylated with 100 mM iodoacetamide in 100 mM TEAB at room temperature for 30 min in the dark. Samples were reduced again with 100 mM DTT in 100 mM TEAB at room temperature for 15 min and subsequently digested with Sequencing Grade Modified Trypsin (Promega, Madison, WI, USA) for 16 hours at 37°C. Samples were dried in a CentriVap DNA Concentrator (Labconco Co., Kansas City, Missouri, USA) and resuspended in 0.1% formic acid (FA; Thermo Fisher Scientific Inc.) for Nano-LC-MS/MS.

### Nanoscale liquid chromatography-tandem mass spectrometry

TRPM6 and TRPM7 proteins were analyzed on a Nano-LC-MS/MS system that included a Nano-liquid chromatograph (Dionex Ultimate 3000, RSLCnano System, Thermo Fisher Scientific Inc.) and a CaptiveSpray source/Quadrupole ion trap mass spectrometer (Model Q-ToF Com-pact II, Bruker, Hamburg, Germany). Peptides were enriched by the Nano trap column and separated on a PepMap100 C18 LC column. Peptides were eluted at a flow rate of 300 nL/min at 60°C under a linear gradient of 2%–95% Solvent B over a 90 min run of mobile phase A. Mobile phase A consisted of water/FA (99.9:0.1, v/v) while solvent B was composed of acetonitrile/water/FA (80:19.92:0.08, v/v). Mass spectral data from the 300 to 2,200 m/z range were collected in the positive ionization mode with acquisition rate set at 6 Hz. Auto MSN CID fragmentation experiments were performed at low (4 Hz) and high (16 Hz) mass spectral rates for the top 2 most intense precursor ions using 3 sec dynamic exclusion. Peptide sequences were matched on the UniProtKB database (https://www.uniprot.org/help/uniprotkb) using the MASCOT (v 2.3) searching engine (Matrix Science Ltd., London, UK). Exponentially modified protein abundance index (emPAI) was used to determine protein abundance in each LC-MS/MS experimental sample (29), while phosphorylation and oxidation of TRPM6 and TRPM7 were determined by MS/MS fragmentation analysis.

### Statistical analysis

Results were expressed as means ± SE. Two sets of data were compared using the unpaired Student’s *t*-test. One-way analysis of variance (ANOVA) with Dunnett’s posttest was used for the comparison of multiple sets of data. All data were analyzed by GraphPad Prism (GraphPad Software Inc., San Diego, CA, USA).

## Results

### Higher membrane TRPM6 expression in small intestine of PPIH rats

**Figure 1:**
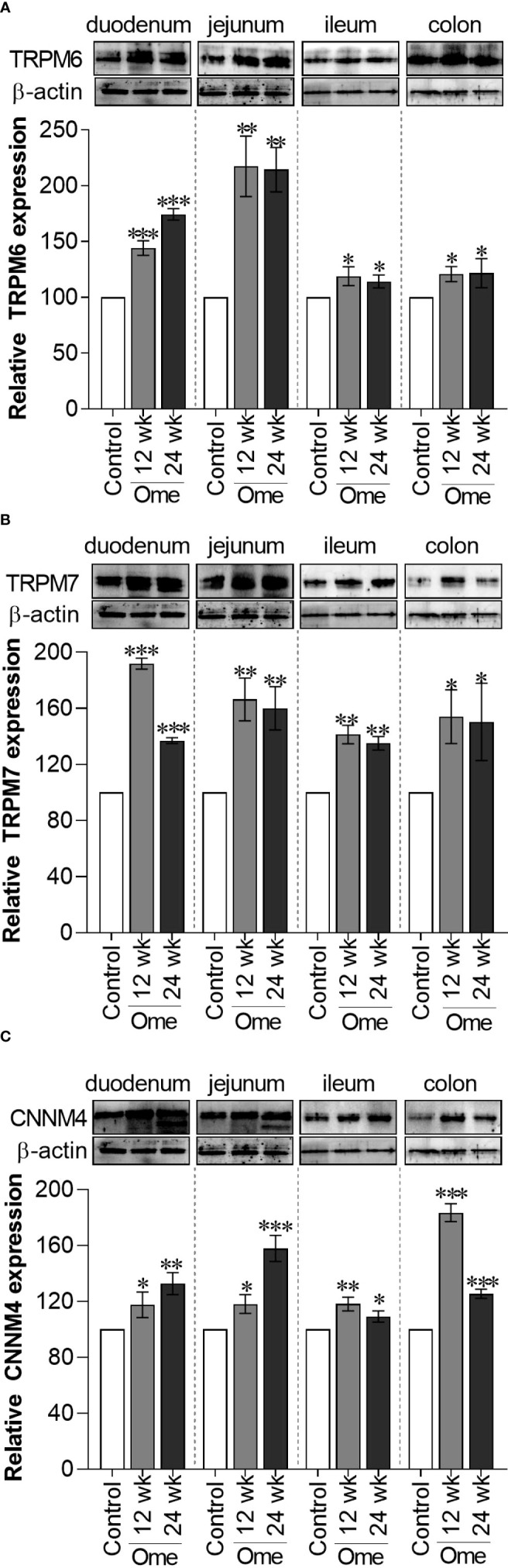
Total TRPM6 **(A)**, TRPM7 **(B)**, and CNNM4 **(C)** expression in duodenum, jejunum, ileum, and colon of control or omeprazole treated rats. ome; omeprazole. *P < 0.05, **P < 0.01, ***P < 0.001. (*n* = 6).

**Figure 2:**
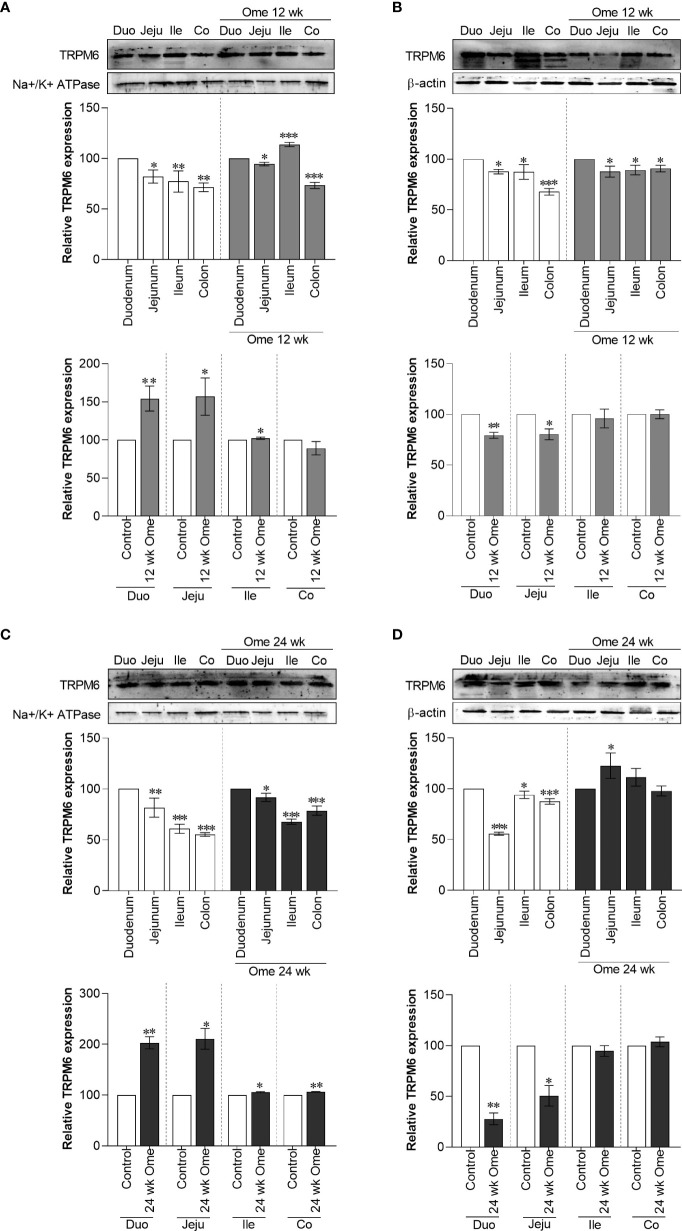
Membrane TRPM6 **(A)** and cytosolic TRPM6 **(B)** expression in duodenum, jejunum, ileum, and colon of control or 12 wk omeprazole-treated rats. Membrane TRPM6 **(C)** and cytosolic TRPM6 **(D)** expression in duodenum, jejunum, ileum, and colon of control or 24 wk omeprazole-treated rats. The upper relative expression graph; the protein expression in jejunum, ileum, and colon was relatively compared to that of in the duodenum. The lower relative expression graph; the protein expression in duodenum, jejunum, ileum, and colon of omeprazole-treated groups was relatively compared its corresponding segment of control group. Duo, duodenum; Jeju, jejunum; Ile, ileum; Co, colon; ome, omeprazole. *P < 0.05, **P < 0.01, ***P < 0.001. (*n* = 6).

In accordance with our previous results (7), both 12-wk and 24-wk omeprazole injection markedly induced total TRPM6 expression in the duodenum, the jejunum, the ileum, and the colon (**Figure 1A**). Compared to controls, membrane TRPM6 expression was significantly higher in the duodenum and the jejunum of 12-wk (**Figure 2A**) and 24-wk omeprazole-injected rats (**Figure 2C**). In contrast, cytosolic TRPM6 was significantly lower in the duodenum and the jejunum of 12-wk (**Figure 2B**) and 24-wk omeprazole-injected rats (**Figure 2D**), indicating an increase in TRPM6 insertion in the plasma membrane of the cells in the duodenum and jejunum of PPIH rats.

### Decrease in membrane TRPM7 expression in small intestine of PPIH rats

**Figure 3:**
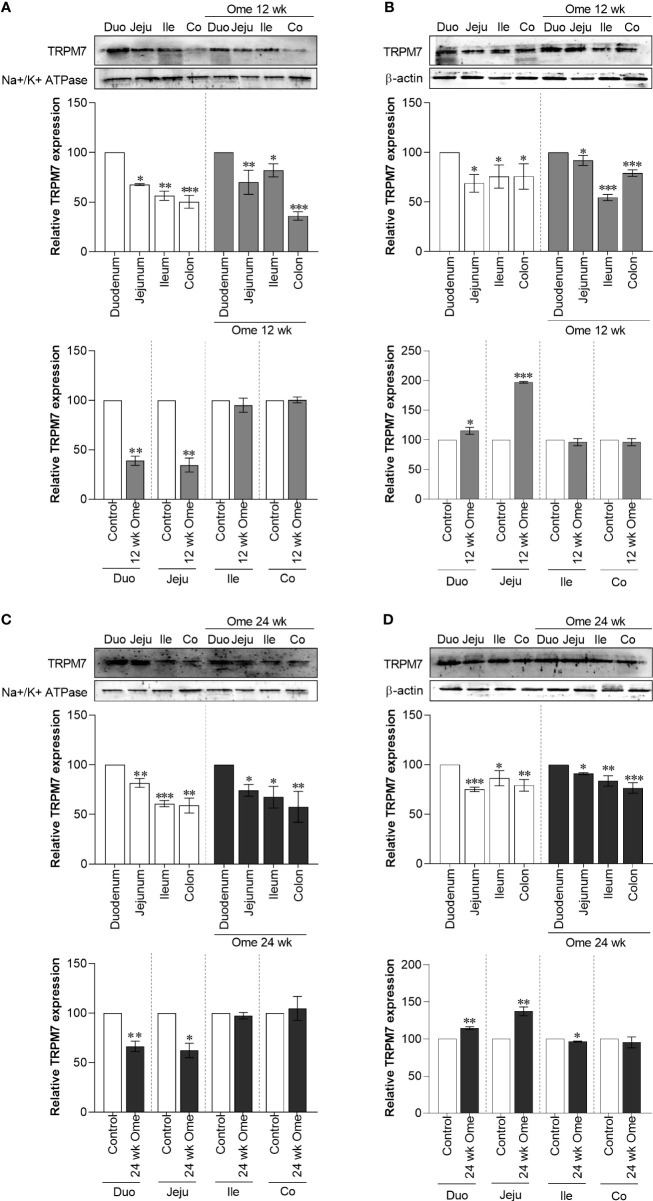
Membrane TRPM7 **(A)** and cytosolic TRPM7 **(B)** expression in duodenum, jejunum, ileum, and colon of control or 12 wk omeprazole-treated rats. Membrane TRPM7 **(C)** and cytosolic TRPM7 **(D)** expression in duodenum, jejunum, ileum, and colon of control or 24 wk omeprazole-treated rats. The upper relative expression graph; the protein expression in jejunum, ileum, and colon was relatively compared to that of in the duodenum. The lower relative expression graph; the protein expression in duodenum, jejunum, ileum, and colon of omeprazole-treated groups was relatively compared its corresponding segment of control group. Duo, duodenum; Jeju, jejunum; Ile, ileum; Co, colon; ome, omeprazole. *P < 0.05, **P < 0.01, ***P < 0.001. (*n* = 6).

As depicted in **Figure 1B**, total TRPM7 had significantly increased in the duodenum, the jejunum, the ileum, and the colon of 12-wk and 24-wk omeprazole-injected rats compared to controls. While membrane TRPM7 in the duodenum and the jejunum of 12-wk (**Figure 3A**) and 24-wk omeprazole-injected rats (**Figure 3C**) had significantly decreased compared to controls, cytosolic TRPM7 expression had significantly increased in the duodenum and the jejunum of omeprazole-treated rats (**Figures 3B, D**). These results suggest that internalization of the plasma membrane TRPM7 probably occurred in the duodenum and the jejunum of PPIH rats.

### Reduction in membrane TRPM6/7 heterodimer in the small intestine of PPIH rats

**Figure 4:**
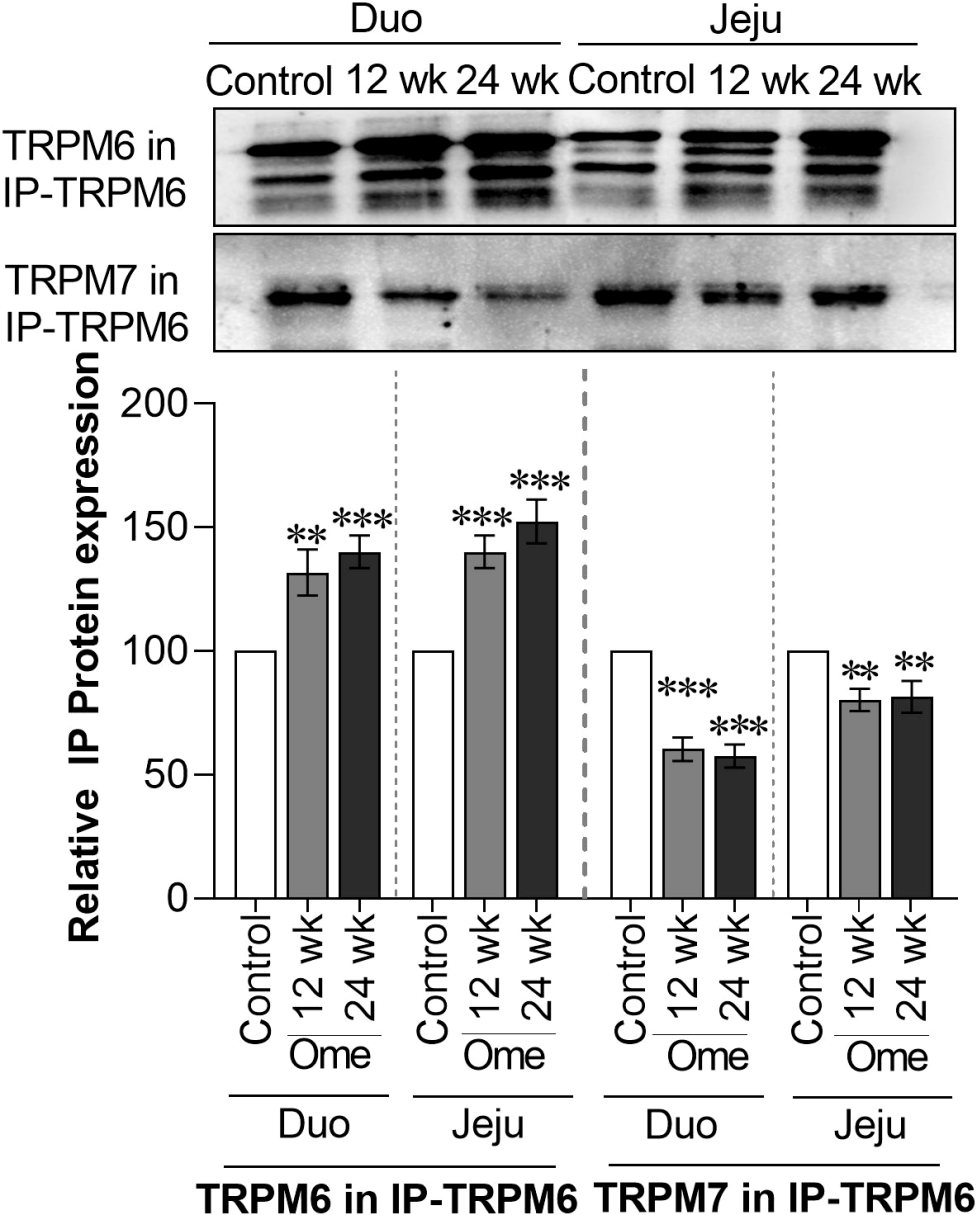
Membrane TRPM6 and TRPM7 expression from immunoprecipitated (IP)-TRPM6 sample from duodenal and ileal tissues of control or omeprazole-treated rats. Duo, duodenum; Jeju, jejunum. **P < 0.01, ***P < 0.001. (*n* = 6).

To analyze the expression of the TRPM6/7 heterodimer, immunoprecipitation of TRPM6 (IP-TRPM6) from the membrane protein fraction was performed. Next, 40 µg of IP-TRPM6 protein from each group was used for western blot analysis. As demonstrated in **Figure 4**, membrane TRPM6 expression from the IP-TRPM6 sample was significantly higher in the duodenum and the jejunum of PPIH rats compared to controls. Western blot membranes were subsequently re-probed with TRPM7 antibody and the results showed that, compared to control rats, TRPM7 expression was significantly lower in IP-TRPM6 samples from the duodenum and the jejunum of omeprazole-injected rats (**Figure 4**). These results indicate lower presence of the membrane TRPM6/7 heterodimer in the duodenum and the jejunum of PPIH rats.

Higher membrane CNNM4 expression in the small intestine of PPIH rats

**Figure 5:**
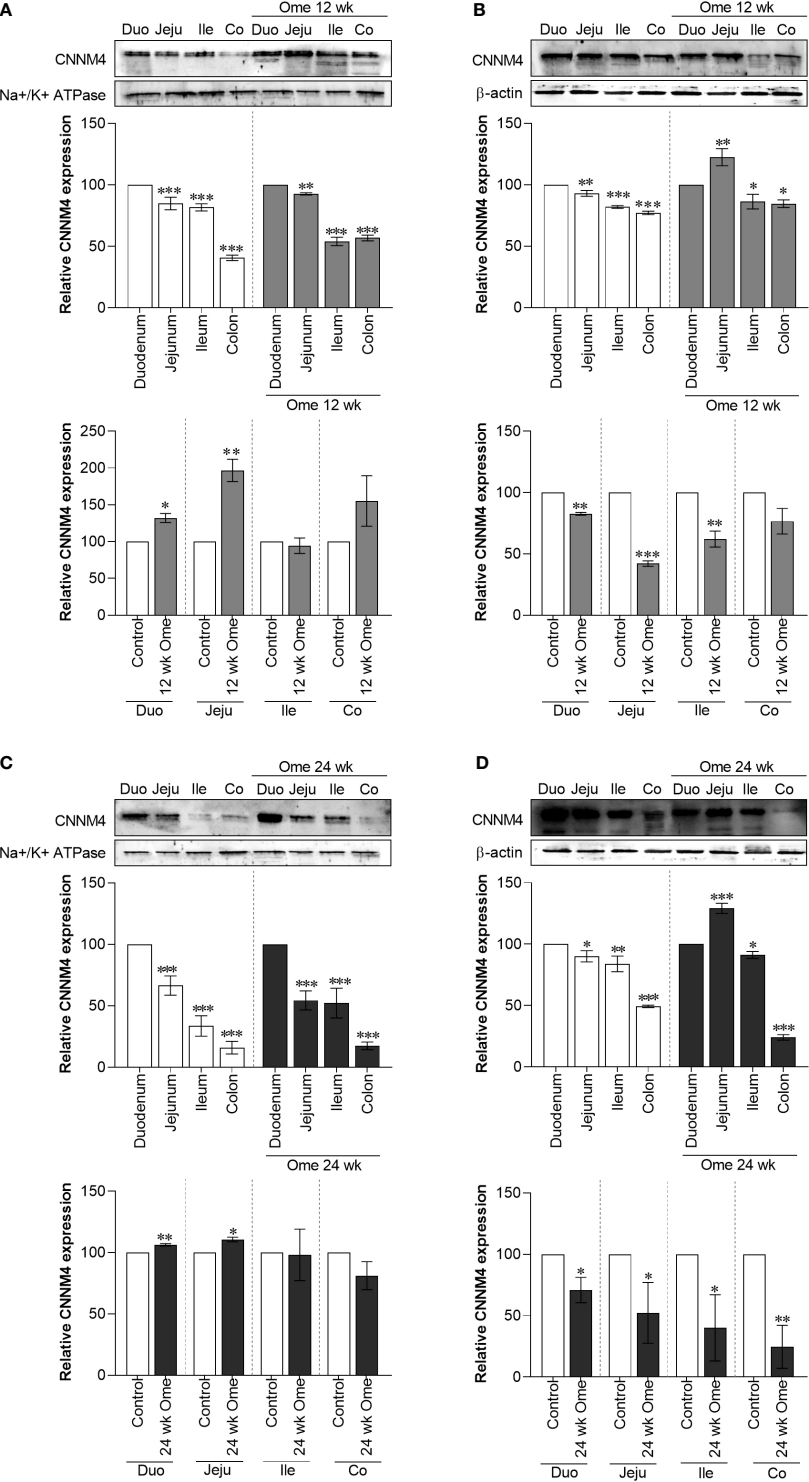
Membrane CNMM4 **(A)** and cytosolic CNMM4 **(B)** expression in duodenum, jejunum, ileum, and colon of control or 12 wk omeprazole-treated rats. Membrane CNMM4 **(C)** and cytosolic CNMM4 **(D)** expression in duodenum, jejunum, ileum, and colon of control or 24 wk omeprazole-treated rats. The upper relative expression graph; the protein expression in jejunum, ileum, and colon was relatively compared to that of in the duodenum. The lower relative expression graph; the protein expression in duodenum, jejunum, ileum, and colon of omeprazole-treated groups was relatively compared its corresponding segment of control group. Duo, duodenum; Jeju, jejunum; Ile, ileum; Co, colon; ome, omeprazole. *P < 0.05, **P < 0.01, ***P < 0.001. (*n* = 6).

As shown in **Figure 1C**, total CNNM4 significantly increased in duodenum, jejunum, ileum, and colon of omeprazole-injected rats compared to controls. Similarly, membrane CNNM4 was significantly higher in the duodenum and the jejunum of 12-wk (**Figure 5A**) and 24-wk omeprazole-treated rats (**Figure 5C**), compared to control animals. Contrastingly, cytosolic CNNM4 expression had significantly decreased in the duodenum and the jejunum of these hypomagnesemic rats (**Figures 5B, D**).

### TRPM6 and TRPM7 protein sequence in duodenum and jejunum of PPIH rats

As our results showed an increase in membrane TRPM6 expression but a reduction in membrane TRPM6/7 heterodimer presence in duodenum and jejunum of PPIH rats, along with marked suppression of Mg^2+^ absorption (7), we further analyzed the protein sequence of plasma membrane-bound TRPM6 and TRPM7 in IP-TRPM6 protein samples using Nano-LC-MS/MS. The emPAI values for IP-TRPM6 from the duodenum and the jejunum of control and omeprazole-treated groups were 0.11 and 0.12, respectively, which indicated that comparable protein quantities had been analyzed by the Nano-LC-MS/MS. TRPM6 protein sequence from the duodenum and the jejunum of control, 12-wk, and 24-wk omeprazole-injected rats showed 98%–100% identity to the human TRPM6 (UniProtKB: Q9BX84), mouse TRPM6 (UniProtKB: Q8CIR4), and a rat non-specific serine/threonine protein kinase, which had probably been translated from the *Trpm6* gene (UniProtKB: F1M7G0) (**Supplement Table 1**). We also analyzed the protein sequence of TRPM7 from the heteromeric TRPM6/7 complex in IP-TRPM6 protein samples from duodenum and jejunum of control and omeprazole (12-wk and 24-wk) treated rats and found that protein sequence had 100% identity to rat TRPM7 (UniProtKB: Q925B3), 99%–100% identity to mouse TRPM7 (UniProtKB; Q923J1), and 99%–100% identity to human TRPM7 (UniProtKB; Q96QT4) sequences (**Supplement Table 2**). These results confirm the presence of membrane TRPM6, TRPM7, and TRPM6/7 heterodimer in the small intestine.

### Phosphorylation of TRPM6 and TRPM7

Next, MS/MS fragmentation analysis was used to identify phosphorylated residues throughout the full length of the TRPM6 and the TRPM7 protein sequence. Previous studies have reported that the S141 residue of TRPM6 regulates the localization and function of membrane TRPM6/7 heterodimer (23), and we detected phosphorylation of the S141 residue in TRPM6 in the duodenum and the jejunum of control rats (**Table 1**). Contrastingly, phospho-S141 was not present in either duodenal or jejunal TRPM6 from PPIH rats, and these observations explain lower membrane expression of the TRPM6/7 heterodimer in the small intestine of our PPIH rat model.

**Table 1:** Phosphorylated residue of TRPM6 protein from duodenum and jejunum of control or omeprazole-treated rats.

	Duodenal TRPM6 protein			Jejunal TRPM6 protein		
	control	12-wk omeprazole	24-wk omeprazole	control	12-wk omeprazole	24-wk omeprazole
**N-terminus**	S12, S67, S78, Y80, T87, T88, S90, T92, T94, S141, T169, T170, T176, T181, Y228, S243, S246, S274, S282, S302, T380, S386, T398, S407, Y431, S445, Y462, Y479, T481, T509, T530, S552, S558, Y574, S586, S596, T602, Y608, T635, S662, Y668, Y697, S705, T706, S743, S750, T756, S820, Y845, T859, S869	S12, S32, T94, Y111, Y116, S153, T169, T170, T176, T181, S184, T204, Y228, T230, S236, T239, Y259, S274, S282, T354, S362, T380, S407, S409, Y431, S445, T471, Y506, T509, Y519, Y525, Y529, T530, Y545, S551, S552, S558, Y574, S586, S590, T602, Y645, S655, Y668, S669, S705, T706, S713, S743, T759, S769, S774, S784, S789, S790, S791, S794, S796, S820, Y845, S869	S12, S15, S28, S32, S33, T40, S67, S71, S82, T87, T88, S90, T92, T94, T97, Y111, T118, S141, T150, S153, S160, T170, T176, S198, T204, T239, T255, S274, T306, T329, S358, T380, S386, T398, T404, S445, Y462, Y479, T481, T488, S504, Y506, T509, Y538, Y542, S551, T562, T570, Y574, S662, Y668, S669, T693, S705, T723, T725, T730, S743, Y783, S784, S794, S796, Y800, T828, Y845, T846, T859	S15, Y31, S78, S82, T87, T88, S90, T92, T94, S141, S160, T169, S198, T204, Y228, T239, T240, S243, S246, S282, S312, T329, T354, S386, T404, Y462, Y479, S527, Y529, Y538, Y542, Y545, S552, S558, S561, T562, S586, S596, S601, S655, S662, T693, S703, S705, T706, S713, S721, T730, T756, T759, S774, S790, Y835, Y845	T28, S32, S33, Y66, S67, T69, S71, S78, T87, T88, S90, Y116, T150, S160, T181, S184, S193, S195, S196, Y228, T230, S236, T239, S274, S282, S362, T380, S386, S407, Y431, T471, Y479, T488, T501, T506, Y519, T530, S551, S552, S558, S561, T562, T570, Y574, S590, T635, Y645, S662, Y668, S669, T693, T706, S743, Y782, Y783, S784, S790, S791, S794, S796, T828, Y849, Y858	S15, T28, S32, S67, T69, S71, S78, T88, T97, T106, Y116, T150, S153, S160, T176, S195, S196, S198, T204, Y228, T240, S243, Y259, Y272, S282, S302, T319, T329, T354, S407, S409, T486, S504, Y506, T509, Y519, Y525, Y530, Y538, S551, S579, S585, S590, S596, S601, T602, Y608, Y645, Y668, S669, T693, T696, Y697, S703, S705, S743, S790, Y800, S820, T828, Y832, Y835, Y845, T857, T859
**Channels**	Y878, 915, T936, Y966, T974, S990, Y1026, S1035,	S876, T881, S893, S907, T913, T915, Y966, T968, T974, Y979, S1008, Y1026, Y1053	S876, Y878, T881, S893, T899, S907, T913, T915, S922, T936, T968, T974, S1034, T1046, Y1053	S876, Y878, T881, S893, T913, T935, Y941, Y966, Y1026	Y878, T881, T899, Y909, T913, T915, T936, S990, 1006, S1008, S1035, S1043, Y1053	T881, S893, T899, T936, S951, T974, S1000, S1006, Y1018, Y1022, S1038, T1046, Y1053
**TRP**	Y1073, S1078, Y1086, Y1089, T1094, Y1095, S1109	Y1073, S1078, S1080, Y1091, Y1095	Y1073, S1078, S1080, Y1086, Y1089,	Y1073, S1078, S1080, Y1086, Y1089, Y1091, T1094, 1095, S1109	Y1073, S1078, T1094, S1109	Y1073, 1078, S1080, T1094, S1109
**C-terminus**	Y1137, S1139, Y1157 S1168	S1139, Y1157, S1168	S1139, S1168	Y1137, S1139, Y1157, S1168	Y1137, Y1157	Y1137, S1168
**Coiled coil**	T1176, S1177, T1181, Y1184, S1195, S1200, S1203, S1206, T1221	T1176, T1181, T1184, S1226	T1176, T1181, S1216 S1225	T1176, S1177, T1181, Y1184, S1195, S1200, S1206, T1221	S1200, S1203, S1206, S1215, T1221	S1200, S1203, S1206, S1215
	T1231, **S1252**, S1281, S1285, T1322, S1324, S1329, S1349, S1357, T1375, T1391, S1395, S1399	T1231, S1244, T1245, S1254, Y1273, S1277, S1281, T1311, S1332, S1339, Y1341, S1368, T1375	T1231, T1240, S1244, T1311, T1322, S1329, S1332, Y1341, S1349, S1357, T1368, S1404	S1226, T1230, S1244, T1245, **S1252**, Y1276, S1285, T1322, S1325, S1329, S1332, Y1341, S1365, S1399	S1226, S1244, S1254, Y1276, T1311, S1325, S1329, S1339, Y1341, S1349, S1357, S1368, S1399, T1426	S1244, Y1275, S1277, S1280, S1281, T1311, T1322, S1324, S1325, S1357, S1368, T1391, S1395, T1426
**S/T rich domain**	S1438, S1441, S1458, T1463, S1487, S1498, S1500, S1503, Y1533, S1539, S1541, T1577, T1598, S1603, S1616, S1618, S1623, T1628, S1630, S1633, T1635, T1647, Y1660, S1672, T1679, S1685, S1689, S1690, S1697, S1699, S1702, T1739, Y1741	T1435, S1437, S1467, T1474, S1478, T1479, S1498, S1500, T1523, Y1533, S1539, S1541, S1562, S1563, T1577, S1583, T1589, S1605, Y1622, S1623, T1628, S1630, S1658, S1664, S1669, S1678, T1679, S1685, S1689, S1690, S1697, S1699, S1702	T1435, S1437, S1458, S1484, S1485, S1487, S1497, S1498, S1513, S1524, Y1533, S1562, S1563, S1583, S1605, S1616, S1618, Y1622, S1623, S1630, Y1640, Y1660, S1669, S1672, S1676, S1678, T1679, S1685, S1697, S1702	Y1452, T1463, S1467, S1498, S1500, S1503, S1509, Y1533, S1541, T1577, T1598, S1618, Y1622, S1623, T1628, S1630, S1633, Y1640, S1658, Y1660, S1664, S1676, S1678, T1679, S1685, S1689, S1690, S1702, T1739, 1741	S1428, T1430, T1435, S1441, T1463, S1467, T1474, S1478, T1479, S1482, S1484, S1485, S1506, S1509, S1510, S1513, S1539, S1562, S1563, S1583, T1589, S1623, S1630, T1635, T1647, S1664, S1685 S1689, S1690	S1428, T1430, T1435, S1441, T1463, S1467, T1474, S1478, T1479, S1485, S1506, S1509, S1510, S1513, S1539, S1563, S1583, S1616, S1618, Y1622, S1623, T1628, S1658, Y1660 S1664, S1669, S1672, S1676, S1678, T1679, S1685, S1689, S1690
**Dimerization motif**	Y1710, S1711, S1722, T1724, T1728, Y1731	Y1710, S1711, T1724 T1728	S1711	Y1710, S1711, S1722, T1724, T1728	Y1710, S1711	Y1710, S1711
	S1736, S1737, T1739, Y1741, S1746, S1747	Y1741	Y1741	S1737, T1739, Y1741, S1746, S1747	S1746, S1747	S1746
**α -Kinase domain**	S1754, S1757, S1821, T1822, T1843, Y1854, Y1878, Y1880, T1895, T1897, S1908, Y1912, Y1914, T1915, T1932, S1935	S1754, S1759, S1771, S1787, S1790, T1813, T1822, T1843, **T1851**, Y1854, T1855, Y1865, S1868, Y1886, S1908, Y1912, Y1914, T1915, S1944	S1754, S1757, S1759, S1771, S1787, T1788, S1790, S1805, T1813, T1822, T1843, **T1851,** Y1854, T1855, Y1865, T1874, Y1878, T1880, Y1886, Y1914, T1915, S1935, S1944	S1754, S1756, S1771, S1787, S1821, T1843, Y1854, Y1865, T1880, Y1886, T1911, Y1914 T1915, S1944	S1754, S1756, S1757, S1759, S1771, S1790, S1788, S1821, T1822, T1843, **T1851**, Y1854, T1855, T1874, Y1878, Y1886, Y1914, T1915, T1932, S1944	S1754, S1756, S1757, S1759, S1771, S1787, T1788, T1813, S1821, T1843, **T1851,** Y1854, T1855, Y1865, T1874, Y1878, T1880, Y1886, Y1914, T1915, S1935, S1944
	S1986, T1993, T2011	S1970, Y1984, T1992, S2002, T2011, S2015	S1970, S2002, T2011, S2015	S1970, Y1985, S1986, T1993, S2002, T2011, S2015	S1970, Y1985, S1986, S1992, T1993, S2002, T2011, S2015	S1970, Y1985, S1986, S2002, T2011, S2015

The highlighted residues have been previously reported to affect TRPM6 activity.

Phosphorylation of the S1252 residue in TRPM6 induces channel permeability (25) and phospho-S1252 was detected in TRPM6 from the duodenum and the jejunum of control rats, but not PPIH rats. Further, T1851 autophosphorylation mediates suppression of TRPM6 channel activity by intracellular free Mg^2+^ and activated C-kinase 1 (RACK1) (16), and we observed phospho-T1851 in TRPM6 from the duodenum and the jejunum of PPIH rats, but not controls (**Table 1**). Notably, these results can, at least in part, explain why TRPM6 overexpression failed to increase small intestinal Mg^2+^ absorption in the PPIH rat model (7).

Hyper-phosphorylation of the N-terminus, channel, and α-kinase domain of TRPM6 was detected in PPIH rats (**Table 1**), while hypo-phosphorylation was discovered in the dimerization motif domain of TRPM6 from PPIH rats. However, the effect of hypo- and hyper-phosphorylation on TRPM6 and its membrane localization and channel permeability, require further study. MS/MS fragmentation analysis of large- and intermediate-size TRPM6 peptide fragments could not identify the presence of S1821 or T1822 residues in duodenal TRPM6 from PPIH rats; however, both residues were present in duodenal samples from control animals. Further, S1821 and T1822 could be detected in a small duodenal TRPM6 peptide fragment in PPIH rats, but its % peak was lower than that of control duodenal TRPM6. Nevertheless, the role of S1821 and T1822 on TRPM6 membrane expression and function require further study.

A previous study has reported that S138 in TRPM7 can modulate its membrane localization (23), and that the S1360 residue in TRPM7 can affect its stability on the plasma membrane (26). In the duodenum and the jejunum of control rats, phospho-S138 and phospho-S1360 residues were detected in TRPM7 (**Table 2**). Neither phospho-S138 nor phospho-S1360 were identified in duodenal or jejunal TRPM7 from PPIH rats, and these results can account for the lower expression of membrane TRPM7 and TRPM6/7 heterodimer in the small intestine of our PPIH model. Hypo-phosphorylation of TRPM7 in the small intestine in PPIH rats was also seen (**Table 2**), and we detected a less phosphorylated residue of TRPM7 that had been phosphorylated by the α-kinase domain of TRPM6 (yellow highlight; **Table 2**).

**Table 2:** Phosphorylated residue of TRPM7 protein from duodenum and jejunum of control or omeprazole-treated rats.

	Duodenal TRPM7 protein			Jejunal TRPM7 protein		
	control	12-wk omeprazole	24-wk omeprazole	control	12-wk omeprazole	24-wk omeprazole
**N-terminus**	S9, T10, T12, Y18, S22, S23, Y62, S63, T55, S57, Y62, S79, T84, S87, T89, S101, S103, Y104, Y108, S113, T115, S138, S193, S196, S233, S243, Y256, T270, Y303, T318, Y327, T332, T349, T353, S359, T379, S385, T397, S406, Y430, S453, S464, T470, Y505, T508, Y528, S539, S547, S552, S554, S561, T564, T583, Y587, T603, T615, S648, S683, T737, T739, S745, T778, S788, S823, S824, S836, Y849	T12, T55, S57, Y62, S63, T84, S87, S101, S103, Y104, Y108, S112, Y113, T115, T173, S193, S195, S196, T201, T227, S233, S243, T252, Y256, T269, Y303, S307, T318, T332, T349, T353, S385, T397, T403, S453, S464, T470, T480, T485, T508, Y524, T529, S552, S554, S561, T564, S594, T603, Y620, S661, S683, S697, T710, Y711, T778, S783, S788, T795, S794, S824 S836	T12, S22, S23, T55, T58, S63, S79, S87, S101, S103, Y104, Y108, S103, T166, T167, T201, S233, S243, T269, S307, T318, Y327, T332, S348, T349, T403, S406, T414, Y430, T480, T523, T528, S539, S552, S561, T564, T583, Y587, S594, T615, Y620, Y659, S661, S669, S697, Y711, S719, T720, S745, Y776, S783, S788, T795, S794, S836	S2, Y18, S22, S23, S63, S74, S79, S87, T89, Y92, S101, S103, Y108, S112, Y113, T115, **S138**, T166, T167, T173, S195, S196, T201, Y225, T227, S243, T252, T269, T318, T332, T353, S359, T367, T379, Y430, S438, S453, S464, T508, S539, S547, S552, S553, S554 T555, S561, T564, T583, Y587, S648, S661, S676, S727, S728, T778, S783, S788, T795, S799, S823, S836, Y849, T860, Y863	Y18, S23, T55, T60, S63, S79, T84, S87, S101, S103, T166, T167, S193, S196, T201, T227, S233, T252, T269, T299, S307, T318, Y327, T332, S348, S385, T414, Y430, S438, S453, Y478, T480, T485, Y505, Y518, Y524, T527, T529, S539, S547, S554, T555, S561, T564, T583, Y587, S648, Y659, S661, S676, Y682, S683, Y711, Y719, S757, Y776, S788, T795, T807, S824, S836	Y18, T55, S57, T60, Y62, S63, T60, Y62, T115, T173, S193, S196, T201, T227, T252, T299, S307, T318, T332, S348, T349, T353, S359, T403, S406, Y430, S438, S453, T470, T485, T508, Y518, Y524, T527, T529, S539, S547, S553, S554, T564, S584, T615, Y620, S648, Y659, S683, S697, T737, T739, T795, S799, T807, S836, T842
**Channels**	Y892, T895, Y896, S913, S921, S927, T929, S934, Y1023, S1031, Y1049, Y1051, S1060, T1064, T1070, T1073, Y1080, Y1085	Y896, T929, S934, Y953, Y965, Y989, Y1002, S1031, Y1049, Y1051, S1060, T1073, Y1080, Y1085	S907, S913, Y949, Y953, S1031, Y1041, Y1049, S1060, T1064, T1070, T1073	S913, S921, S929, Y949, Y953, Y989, Y1002, S1013, Y1023, S1029, S1031, Y1041, Y1051, S1060, T1064, Y1080, Y1085	S883, S907, S913, S927, Y949, Y953, Y973, S1013, Y1023, S1029, Y1041, S1060, T1070, T1073	Y923, T929, S934, Y953, Y965, Y973, Y989, S1029, Y1051, T1064, T1073, Y1080
**TRP**	Y1100, S1107, Y016, Y1122, S1036, S1040, S1141	Y1100, S1107, Y1016, Y1122, S1036, S1040	Y1100, S1107, S1036, S1040	Y1100, S1107, Y1116, S1136, S1140	S1107, Y1113, Y1116	S1136
**C-terminus**	T1154, S1155, T1163, Y1181, S1191	S1155, T1163, Y1181	T1154, S1155	S1155, T1163, Y1181, S1191, S1193	S1155, T1163, S1193	S1155
**Coiled coil**	T1200, S1208, S1224, T1242, T1245, T1248, T1250	T1200, S1208, Y1220, S1224, S1227, S1230, T1245	S1208, Y1220, S1224, S1230, T1242, T1245	T1200, Y1220, S1224, S1227, S1230, S1239, T1242, T1250	T1200, S1208, Y1220, S1224, S1227, S1230, T1245	S1227, T1245, T1248, T1250
	S1255, S1258, T1265, S1269, S1271, S1292, T1296, S1298, S1299, S1300, S1308, S1349, S1350, **S1360**	S1255, S1300, Y1326, S1349, S1350, S1357, S1385	S1255, S1258, T1265, S1292, S1357	S1255, S1258, S1271, S1292, T1296, S1298, S1299, S1300, S1308, S1349, S1350, S1357, **S1360**	S1258, T1265, S1269, S1271, S1298, S1299, S1300, S1308, Y1326, S1357	S1258, S1299, S1300, S1349, S1350, S1357
**S/T rich domain**	S1386, S1389, S1390, S1394, S1395, S1403, T1404, S1406, S1409, S1412, S1416, T1418, S1445, S1455, S1463, T1466, T1470, Y1479, T1485, T1487, S1488, S1491, T1493, S1495, S1497, S1501, T1502, S1505, S1510, T1524, S1530, T1534, S1540, T1548	S1385, S1386, S1389, S1390, S1395, S1406, S1409, S1403, T1404, S1406, S1409, S1412, T1417, T1418, S1445, T1454, S1455, S1463, T1470, T1485, S1510, S1530, S1540, T1548	S1385, S1386, S1389, S1390, S1394, S1403, T1404, S1406, S1409, T1418, S1445, T1454, S1455, S1463, T1466, S1491, T1493, S1495, S1530, T1534	S1386, S1389, S1390, S1394, S1395, S1403, S1406, S1409, S1412, S1416, T1418, Y1426, S1445, T1454, S1455, T1466, T1470, Y1479, T1485, T1487, S1488, S1491, T1493, S1495, S1497, S1501, T1502, S1505, S1510, T1524, S1530, T1534, S1540, T1548	S1386, S1389, S1390, S1394, S1395, T1398, S1401, S1403, T1404, T1418, T1493, S1505, S1510, T1524, S1530, T1534, S1540, T1548	S1390, S1394, S1395, S1406, S1409, S1412, T1454, T1485, S1488, T1493, S1495, S1497, S1501, T1502, T1524
**Dimerization motif**	S1553, S1564, S1566	S1553, S1564, S1566	S1553, S1564, S1566	Y1552, S1564, S1566	Y1552, S1564, S1566	Y1552, S1564, S1566
	S1587, S1888, S1590	Y1582, S1590	Y1582, S1590	T1580, S1587, S1588, S1590	S1587, S1588	Y1582, S1588
**α -Kinase domain**	S1595, S1597, S1598, S1600, S1612, T1629, S1631, Y1642, S1656, S1657, Y1659, T1663, S1692, Y1695, Y1696, S1709, Y1727, T1738, T1740, S1749, T1756, S1776, S1785	S1595, S1597, S1598, S1612, T1629, S1631, S1638, Y1642, S1656, S1657, Y1659, T1663, T1682, Y1695, S1696, Y1706, S1709, T1721, S1749, T1752, S1785	S1595, S1597, S1598, T1629, Y1642, S1656, S1657, Y1659, T1663, Y1695, S1709, T1740, T1752, T1756, S1785	S1592, S1598, S1600, S1612, T1629, S1638, S1646, S1656, Y1659, T1663, T1682, S1692, Y1695, S1709, T1740, S1749, T1752, Y1753, Y1755, T1756, S1776, S1782, S1811	S1598, S1595, S1597 , S1598, T1629, Y1642, S1656, S1657, Y1659, T1663, Y1695, S1709, T1740, T1752, T1756, S1785	S1598, T1629, S1631, S1631, S1646, T1663, Y1695, S1696, S1749, T1752, T1773, S1776, S1785
	Y1826, T1827, S1839, S1848, T1849, T1855	Y1826, T1827, S1839, T1849, T1855	T1827, S1839,	T1827, S1839, S1848, T1849, S1852, S1857	T1827, S1838, S1839, S1857	T1827, S1838, S1839

The highlighted residues have been previously reported to affect TRPM6 activity.

### Hyper-oxidation of TRPM6 and TRPM7

MS/MS fragmentation analysis also revealed methionine oxidation throughout the full length of the TRPM6 (**Table 3**) and the TRPM7 (**Table 4**) protein sequences. We also identified hyper-oxidation of TRPM6 and TRPM7 protein in the small intestine of PPIH rats compared to control rats. A previous study has reported that oxidation of the M1755 residue leads to the suppression of TRPM6 channel activity (27), but as seen in **Table 3**, M1755 oxidation (bolded) was present in both duodenal and jejunal TRPM6 in PPIH rats, but not in control rats. These results can, at least in part, explain why TRPM6 overexpression fails to increase small intestinal Mg^2+^ absorption (7). Importantly, our results suggest that prolonged omeprazole treatment increases oxidative stress in the small intestine.

**Table 3:** Methionine oxidation in TRPM6 protein from duodenum and jejunum of control or omeprazole-treated rats.

	control	12-wk omeprazole	24-wk omeprazole
**duodenum**	M33, M63, M133, M338, M370, M416, M618, M623, M625, M648, M657, M732, M768, M847, M864, M969, M973, M984, M1020, 1061, M1076, M1093, M1162, M1278, M1183, M1434, M1436, M1575, M1879, M2020	M1, M33, M63, M133, M151, M244, M338, M370, M373, M416, M444, M450, M618, M623, M625, M648, M657, M692, M727, M732, M734, M739, M768, M780, M847, M969, M984, M1076 M1093, M1162, M1183, M1190, M1265, M1434, M1436, M1551, M1575, M1645, M1719, **M1755**, M1879, M1904, M1947, M2020	M1, M33, M63, M127, M133, M151, M244, M263, M350, M373, M416, M444, M450, M618, M623, M625, M648, M657, M692, M727, M732, M734, M739, M768, M780, M847, M854, M864, M969, M984, M1020, M1061, M1076, M1093, M1183, M1190, M1446, M1551, M1645, M1719, **M1755**, M1775, M1879, M1904, M2020,
**jejunum**	M127, M133, M151, M263, M338, M354, M370, M734, M739, M780, M864, M969, M977, M984, M1183, 1190, M1265, M1278, M1434, M1436, M1446, M1551, M1645, M1719, M1766, M1947, M2020	M1, M127, M133, M151, M244, M263, M350, M370, M373, M416, M450, M618, M625, M648, M657, M692, M727, M732, M734, M847, M854, M864, M969, M1076, M1093, M1162, M1183, M1434, M1446, M1551, M1575, M1645, M1719, **M1755**, M1766, M1775, M1783, M1879, M2020	M1, M127, M133, M151, M244, M263, M338, M350, M370, M416, M444, M450, M618, M625, M648, M657, M692, M727, M734, M768, M847, M864, M973, M977, M984, M1020, M1061, M1076, M1093, M1162, M1183, M1265, M1434, M1436, M1645, M1719, **M1755**, M1766, M1783, M1879, M1904, M1947, M2020

The highlighted residues have been previously reported to affect TRPM6 activity.

**Table 4:** Methionine oxidation in TRPM7 protein from duodenum and jejunum of control or omeprazole-treated rats.

	control	12-wk omeprazole	24-wk omeprazole
**duodenum**	M43, M372, M595, M632, M637, M649, M706, M742, M746, M782, M794, M812, M868, M878, M906 M991, M996, M1000, M1088, M1020, M1180, M1207, M1287, M1319, M1373, M1446, M1528, M1616, M1720, M1745	M130, M143, M369, M372, M449, M466, M575, M591, M595, M649, M662, M704, M706, M741, M746, M773, M782, M812, M830, M868, M991, M992, M996, M1000, M1043, M1180, M1207, M1287, M1318, M1373, M1446, M1528, M1561, M1596, M1616, M1720, M1745, M1788	M1, M130, M130, M143, M369, M372, M449, M465, M466, M575, M591, M649, M662, M704, M741, M753, M773, M782, M794, M796, M812, M830, M868, M878, M991, M992, M1007, M1043, M1120, M1207, M1287, M1373, M1393, M1446, M1528, M1561, M1596, M1688, M1720, M1745, M1788, M1891
**jejunum**	M130, M369, M372, M449, M465, M649, M662, M704, M706, M741, M746, M796, M812, M830, M991, M996, M1000, M1088, M1180, M1373, M1446, M1528, M1561, M1616, M1688, M1720, M1788	M1, M130, M372 M465, M520, M575, M591, M595, M649, M662, M741, M773, M782, M512, M830, M878, M992, M1000, M1043, M1088, M1120, M1207, M1287, M1318, M1373, M1393, M1446, M1528, M1561, M1596, M1616, M1688, M1720, M1745, M1788	M1, M43, M130, M143, M449, M465, M488, M520, M575, M591, M595, M632, M637, M649, M662, M748, M753, M782, M794, M796, M812, M830, M878, M906, M992, M1000, M1007, M1043, M1180, M1207, M1287, M1318, M1373, M1393, M1561, M1596, M1616, M1720, M1745, M1788, M1861

## Discussion

We have previously demonstrated that prolonged omeprazole administration (12 and 24 wks) induced hypomagnesemia and Mg^2+^-store depletion by suppressing intestinal Mg^2+^ absorption, mainly in the duodenum and the jejunum of PPIH rats (7). In continuation, here, we show that the expression of the membrane TRPM6/7 heterodimer and that of membrane TRPM7 was markedly lower in the duodenum and the jejunum of PPIH rats, but that membrane TRPM6 expression was higher. Mass spectrometric analysis demonstrated hyper-phosphorylation, especially at S1252 and T1851, and hyper-oxidation at M1755, both of which suppressed membrane TRPM6 channel permeability. Additionally, in PPIH animals, hypo-phosphorylation at S138 and S1360 in TRPM7 disrupted its membrane stability and hyper-oxidation of TRPM7 was observed. These results help explain the mechanism underlying disruption of transcellular Mg^2+^ absorption in the small intestine of PPIH rats. However, we could not assess how omeprazole suppressed paracellular Mg^2+^ absorption in small intestine during PPIH; nevertheless, it is possible that Claudin (Cldn)-16 and -19, which mediate paracellular Mg^2+^ reabsorption in renal tubule (30), are involved. However, the small intestine only expresses Cldn-1, -2, -3, -4, -5, -7, -8, -12, and -15, but not -16 and -19 (31); thus, processes involving Cldn-regulated paracellular Mg^2+^ absorption in the small intestine remain to be elucidated.

We demonstrate the presence of the TRPM6/7 heterodimer in the duodenal and jejunal epithelial plasma membrane, and to the best of our knowledge, this is the first study to do so. The TRPM6/7 channel allows continuous intestinal Mg^2+^ absorption (17, 22), and a reduction in the expression of membrane TRPM6/7 can lead to lower transcellular Mg^2+^ absorption (7). It is known that mutations in S141 in TRPM6 and S138 in TRPM7 abolish membrane TRPM6/7 heterodimer expression (23), and we found no phosphorylation at S141 in TRPM6 and at S138 in TRPM7 from the duodenum and the jejunum of PPIH rats even though phospho-S141 and phospho-S138 in TRPM6 and TRPM7, respectively, were present in control rats. Further, the stability of membrane TRPM7 is regulated by phosphorylation at S1360 (26) but phospho-S1360 was detected only in control animals and not in PPIH rats. Additionally, the dimerization motif domain of TRPM6 from the duodenum and the jejunum of PPIH rats displayed lower phosphorylation. Together, these observations, can at least in part, explain the reduction in membrane TRPM6/7 heterodimer presence in the small intestine of PPIH rats.

Our results also describe the presence of membrane-bound and cytosolic TRPM6 expression in the small intestine with duodenal, jejunal, and ileal membrane TRPM6 expression markedly increasing in PPIH rats compared to controls. Hyper-phosphorylation at T1851 in membrane TRPM6 in PPIH rats was also observed, which is essential for the inhibitory effect of intracellular Mg^2+^ and RACK1 on TRPM6 channel activity (16). As RACK1 is extensively expressed throughout the small intestine (32), membrane TRPM6 in PPIH rats was prone to inhibition by intracellular Mg^2+^ and RACK1, which could have led to a decrease in transcellular Mg^2+^ absorption (7). We also show that duodenal, jejunal, and ileal cytosolic TRPM6 expression was clearly lower in PPIH animals compared to controls, but mechanisms contributing to greater plasma membrane expression of TRPM6 in the small intestine of PPIH rats are currently not known. We also show phosphorylation of various serine, threonine, and tyrosine residues in the TRPM6 protein, but the effects of such phosphorylation on channel activity, membrane expression, or TRPM6/7 heterodimerization require further study.

Hyper-oxidation of methionine residues in TRPM6 and TRPM7 was observed in PPIH rats, and it is known that methionine oxidation depends on pH; specifically, a higher pH leads to greater oxidation (33). We have previously reported that omeprazole injection significantly increases luminal pH in the duodenum, the jejunum, and the ileum (7); thus, it is possible that hyper-oxidation of methionine in TRPM6 and TRPM7 of PPIH rats is facilitated by the higher luminal pH. Moreover, prolonged omeprazole administration induced hypomagnesemia, chronic small intestinal inflammation, and villous atrophy (34), and hyper-oxidation of TRPM6 in PPIH rats might have been induced by chronic small intestinal inflammation. Interestingly, oxidation of the M1755 residue in TRPM6 was seen in the duodenum and the jejunum of only PPIH rats and not controls. Thus, it is possible that TRPM6 channel activity was markedly suppressed upon oxidation at M1755 (27), which then led to disruption of transcellular Mg^2+^ absorption in the small intestine of our PPIH rat model (7).

The TRPM6 kinase domain can phosphorylate serine and threonine residues in TRPM7, but not vice versa (35, 36), TRPM6 α-kinase also regulates TRPM7 intracellular trafficking (35), and the active α-kinase for TRPM6 can suppress membrane TRPM6/7 and TRPM7 expression (35) even though a mutant TRPM6 α-kinase promotes membrane TRPM6/7 and TRPM7 expression (35). Here, membrane TRPM7 was markedly decreased in PPIH while cytosolic TRPM7 was significantly increased, and hyper-phosphorylation of the α-kinase domain of TRPM6 was seen in PPIH, which might induce its kinase activity (37). Thus, high TRPM6 kinase activity in PPIH rats probably induced internalization of membrane TRPM7.

CNNM4 mediates basolateral Mg^2+^ extrusion and is implicated in small intestinal transcellular Mg^2+^ absorption (38). Recently, fibroblast growth factor-23 (FGF-23) has been reported to systemically and directly increase membrane CNNM4 expression in the duodenum and the jejunum (12) and our PPIH rat model shows significantly higher plasma FGF-23 compared to controls (12). Therefore, the observed increase in membrane CNNM4 in the duodenum and the jejunum of PPIH rats may involve an FGF-23-dependent mechanism.

Prolong PPI induced gastrointestinal cancer and hypomagnesemia (1–5, 7, 8). Dysregulation of systemic Mg^2+^ homeostasis induced risk of cancer (5, 39). Previous reports revealed that dysregulation of Mg^2+^ channels involved in the regulation of numerous hallmarks of cancer cells, including sustained proliferation, enhanced survival, angiogenesis, and invasion and metastasis (3, 39). In the present study we reported the change in intestinal TRPM6, TRPM7, and TRPM6/7 expression and function in PPIH rats. However, the role of TRPM6, TRPM7, and TRPM6/7 induced risk of cancer in PPIH model requires further study.

To summarize, we confirm the presence of TRPM6, TRPM7, and TRPM6/7 heterodimer in the small intestine of rats and show that prolonged PPI treatment induces hyper-phosphorylation and hyper-oxidation of TRPM6, but hypo-phosphorylation of TRPM7, which then lowers membrane TRPM7 and TRPM6/7 expression and TRPM6 channel permeability, thereby suppressing small intestinal Mg^2+^ absorption in PPIH.

## Data availability statement

The datasets presented in this study can be found in online repositories. The names of the repository/repositories and accession number(s) can be found in the article/**Supplementary Material**.

## Ethics statement

The animal study was reviewed and approved by Ethics Committee on Animal Experiments, Burapha University, Thailand (IACUC 017/2562).

## Author contributions

NK designed and performed experiments, analyzed the results, and wrote the manuscript. NT designed and performed experiments, analyzed and interpreted the results, and wrote and edited the manuscript. The authors declare that all data were generated in-house and that no paper mill was used. All authors contributed to the article and approved the submitted version.

## Funding

This work was funded by Burapha University and Thailand Science Research and Innovation (TSRI) (Grant no. 42/2565) to NT and National Research Council of Thailand (NRCT) (Grant no. M003/2564) to NK.

## Acknowledgments

We express our gratitude to Ms. Punnisa Kulwong of the Faculty of Allied Health Sciences, Burapha University, Ms. Nasisorn Suksridechacin of Biodiversity Research Centre, Thailand Institute of Scientific and Technological Research, Pathum Thani, and Mrs. Supitcha pannengpetch of Center for Research and Innovation, Faculty of Medical Technology, Mahidol University for their excellent technical assistance.

## Conflict of interest

The authors declare that the research was conducted in the absence of any commercial or financial relationships that could be construed as a potential conflict of interest.

## Publisher’s note

All claims expressed in this article are solely those of the authors and do not necessarily represent those of their affiliated organizations, or those of the publisher, the editors and the reviewers. Any product that may be evaluated in this article, or claim that may be made by its manufacturer, is not guaranteed or endorsed by the publisher.
