# Supplementary material for: Mass spectrometric analysis of TRPM6 and TRPM7 from small intestine of omeprazole-induced hypomagnesemic rats
Source: Front Oncol. 2022 Aug 29;12:947899. doi: 10.3389/fonc.2022.947899 (PMC9468766; doi:10.3389/fonc.2022.947899)
Supplement: Supplementary file 1 [file Table_1.docx]

**Supplement Table 1. TRPM6 protein sequence identity.**

| **Groups** | **UniProtKB database** | **% Matching** |
| --- | --- | --- |
| **Duodenum: TRPM6** | | |
| **Control rats** | Human TRPM6 (UniProtKB: Q9BX84) | 99% |
|  | Rat non-specific S/T protein kinase (UniProtKB: F1M7G0) | 99% |
|  | Mouse TRPM6 (UniProtKB: Q8CIR4) | 99% |
| **12-wk omeprazole injected rats** | Human TRPM6 (UniProtKB: Q9BX84) | 98% |
|  | Rat non-specific S/T protein kinase (UniProtKB: F1M7G0) | 99% |
|  | Mouse TRPM6 (UniProtKB: Q8CIR4) | 99% |
| **24-wk omeprazole injected rats** | Human TRPM6 (UniProtKB: Q9BX84) | 98% |
|  | Rat non-specific S/T protein kinase (UniProtKB: F1M7G0) | 99% |
|  | Mouse TRPM6 (UniProtKB: Q8CIR4) | 99% |
| **Jejunum: TRPM6** | | |
| **Control rats** | Human TRPM6 (UniProtKB: Q9BX84) | 99% |
|  | Rat non-specific S/T protein kinase (UniProtKB: F1M7G0) | 100% |
|  | Mouse TRPM6 (UniProtKB: Q8CIR4) | 99% |
| **12-wk omeprazole injected rats** | Human TRPM6 (UniProtKB: Q9BX84) | 99% |
|  | Rat non-specific S/T protein kinase (UniProtKB: F1M7G0) | 99% |
|  | Mouse TRPM6 (UniProtKB: Q8CIR4) | 99% |
| **24-wk omeprazole injected rats** | Human TRPM6 (UniProtKB: Q9BX84) | 99% |
|  | Rat non-specific S/T protein kinase (UniProtKB: F1M7G0) | 99% |
|  | Mouse TRPM6 (UniProtKB: Q8CIR4) | 99% |
