# Supplementary material for: Mass spectrometric analysis of TRPM6 and TRPM7 from small intestine of omeprazole-induced hypomagnesemic rats
Source: Front Oncol. 2022 Aug 29;12:947899. doi: 10.3389/fonc.2022.947899 (PMC9468766; doi:10.3389/fonc.2022.947899)
Supplement: Supplementary file 2 [file Table_2.docx]

**Supplement Table 2. TRPM7 protein sequence identity.**

| **Groups** | **UniProtKB database** | **% Matching** |
| --- | --- | --- |
| **Duodenum: TRPM7** | | |
| **Control rats** | Human TRPM7 (UniProtKB: Q96QT4) | 99% |
|  | Rat TRPM7 (UniProtKB: Q925B3) | 100% |
|  | Mouse TRPM7 (UniProtKB: Q923J1) | 100% |
| **12-wk omeprazole injected rats** | Human TRPM7 (UniProtKB: Q96QT4) | 100% |
|  | Rat TRPM7 (UniProtKB: Q925B3) | 100% |
|  | Mouse TRPM7 (UniProtKB: Q923J1) | 100% |
| **24-wk omeprazole injected rats** | Human TRPM7 (UniProtKB: Q96QT4) | 99% |
|  | Rat TRPM7 (UniProtKB: Q925B3) | 99% |
|  | Mouse TRPM7 (UniProtKB: Q923J1) | 99% |
| **Jejunum: TRPM7** | | |
| **Control rats** | Human TRPM7 (UniProtKB: Q96QT4) | 100% |
|  | Rat TRPM7 (UniProtKB: Q925B3) | 100% |
|  | Mouse TRPM7 (UniProtKB: Q923J1) | 100% |
| **12-wk omeprazole injected rats** | Human TRPM7 (UniProtKB: Q96QT4) | 100% |
|  | Rat TRPM7 (UniProtKB: Q925B3) | 100% |
|  | Mouse TRPM7 (UniProtKB: Q923J1) | 100% |
| **24-wk omeprazole injected rats** | Human TRPM7 (UniProtKB: Q96QT4) | 100% |
|  | Rat TRPM7 (UniProtKB: Q925B3) | 100% |
|  | Mouse TRPM7 (UniProtKB: Q923J1) | 100% |
